# Characterization of large deletions of the *MECP2* gene in Rett syndrome patients by gene dosage analysis

**DOI:** 10.1002/mgg3.793

**Published:** 2019-06-17

**Authors:** Silvia Vidal, Ainhoa Pascual‐Alonso, Marc Rabaza‐Gairí, Edgar Gerotina, Nuria Brandi, Paola Pacheco, Clara Xiol, Mercè Pineda, Rett Working Group, Judith Armstrong

**Affiliations:** ^1^ Sant Joan de Déu Research Foundation Barcelona Spain; ^2^ Sant Joan de Déu Research Institute (IRSJD) Hospital Sant Joan de Déu Esplugues de Lobregat (Barcelona) Spain; ^3^ Facultat de Medicina Universitat de Barcelona Barcelona Spain; ^4^ Molecular and Genetics Medicine Section Hospital Sant Joan de Déu Barcelona Spain; ^5^ CIBER‐ER (Biomedical Network Research Center for Rare Diseases) Instituto de Salud Carlos III Madrid Spain

**Keywords:** large deletions, *MECP2*, Phenotype‐genotype correlations, Rett syndrome

## Abstract

**Background:**

Rett syndrome (RTT) is a developmental disorder with an early onset and X‐linked dominant inheritance pattern. It is first recognized in infancy and is seen almost always in girls, but it may be seen in boys on rare occasions. Typical RTT is caused by de novo mutations of the gene *MECP2* (OMIM*300005), and atypical forms of RTT can be caused by mutations of the *CDKL5* (OMIM*300203) and *FOXG1* (OMIM*164874) genes.

**Methods:**

Approximately 5% of the mutations detected in *MECP2* are large rearrangements that range from exons to the entire gene. Here, we have characterized the deletions detected by multiplex ligation‐dependent probe amplification (MLPA) in the gene *MECP2* of 21 RTT patients. Breakpoints were delineated by DNA‐qPCR until the amplification of the deleted allele by long‐PCR was possible.

**Results:**

This methodology enabled us to characterize deletions ranging from 1,235 bp to 85 kb, confirming the partial or total deletion of the *MECP2* gene in all these patients. Additionally, our cases support the evidence claiming that most of these breakpoints occur in some restricted regions of the *MECP2* gene.

**Conclusion:**

These molecular data together with the clinical information enable us to propose a genotype–phenotype correlation, which is essential for providing genetic counseling.

## 1 INTRODUCTION

Rett syndrome (RTT; OMIM#312750) is a neurodevelopmental disorder with early onset that is most often found in girls. It is first recognized in infancy; a period of apparently normal development (up to the age of 6–18 months) is followed by a stagnation‐regression characterized by a loss of purposeful hand use and speech, motor apraxia that may be associated with epilepsy and dysautonomic features, including disturbed breathing, sleep, and gastrointestinal motility(Hagberg, Aicardi, Dias, & Ramos, 1983). RTT has a worldwide incidence of 1:10,000 live female births and is the second leading cause of severe mental retardation in females.

Since 1999, numerous reports have supported the evidence that mutations in the *Methyl CpG binding protein 2* gene (*MECP2*; OMIM*300005) are the primary cause of classic RTT (Amir et al., 1999). MeCP2 is a transcriptional regulatory protein, and in its absence, a large number of genes exhibit abnormal expression with implications in the balance between synaptic excitation and inhibition (Kron et al., 2012).

The *MECP2* gene is localized in Xq28, contains four exons, and encodes two major functional domains namely: the methyl binding domain (MBD) (Nan, Meehan, & Bird, 1993) and the transcription repression domain (TRD), which contains a nuclear localization signal (NLS) (Singh, Saxena, Christodoulou, & Ravine, 2008). The *MECP2* translational initiation site was originally identified in exon 2, but a second translation initiation site was described in exon 1, which led to a new MeCP2 isoform (Mnatzakanian et al., 2004). MeCP2E1 is comprised of the exons 1, 3, and 4 while MeCP2E2 contains exons 2, 3, and 4; both forms comprise the MBD and TRD domains. MeCP2E1 is much more abundant in the brain while MeCP2E2 has a higher transcriptional expression level in the skeletal muscles, placenta, liver, and prostate gland (Liyanage & Rastegar, 2014).

No clear phenotype–genotype correlation has been identified in RTT patients (Bebbington et al., 2012; Neul et al., 2008; Scala et al., 2007). It has been reported that 95% of individuals affected by classic RTT have a loss of function in *MECP2*, but is less frequently seen in atypical RTT (Neul et al., 2010). There are eight common mutations of this gene that constitute approximately two‐thirds of all mutations. Another small number of the patients carry a deletion ranging between 1 and 338 bp, in the C‐terminal region (see RettBASE; http://mecp2.chw.edu.au/).

Soon after *MECP2* was identified as causative of RTT, several groups started to study the gene dosage of the cases that were negative for point mutations or small *indels* in the coding sequence of the gene. Thus, Southern Blot or MLPA followed by qPCR, long‐PCR, and Sanger sequencing to narrow down the rearrangement, proved to be helpful in explaining approximately 10% of the mutations in those cases (Archer et al., 2006; Erlandson et al., 2003; Laccone et al., 2004; Ravn et al., 2005; Yaron et al., 2002). At our hospital, when taking into account all the cases diagnosed as RTT that have a mutation in *MECP2*, 4.5% of them have large rearrangements (Vidal et al., 2017), which is consistent with what has been reported in the literature (Hardwick et al., 2007).

Here, we present the molecular characterization of the breakpoints of the deletions detected in *MECP2* by MLPA in 21 RTT patients. The patients' clinical information was gathered as well, when available, in order to assess their severity with Pineda's score to determine a genotype–phenotype correlation and attempt to improve the genetic counseling for these and similar families.

## 2 MATERIALS AND METHODS

### 2.1 Patients and DNA samples

#### 2.1.1 Ethical compliance

Written informed consent was obtained from individuals legally responsible for the patients in accordance with appropriate ethics protocols for the analysis of genes related to RTT.

This study involved 21 patients clinically diagnosed with classic RTT who were negative for *MECP2* point mutations and small *indels* in the coding sequence. To evaluate the severity of the clinical presentation of each patient, a set of symptoms were measured using the clinical severity scores designed by Dr. Pineda (Monrós et al., 2001).

DNA was extracted from peripheral blood leukocytes using the Puregene DNA Isolation kit (Gentra System, Minneapolis, USA).

### 2.2 MLPA analysis

All patients were analyzed by MLPA. MECP2‐MLPA was performed with SALSA P015‐D1, P015‐E1 or P015‐F1 kits (MRC‐Holland, Amsterdam, The Netherlands) in accordance with the manufacturer's instructions. This assay covers all four *MECP2* exons and the flanking genes *IRAK1* (OMIM*300283), *L1CAM* (OMIM*308840), and *VAMP7* (OMIM*300053).

### 2.3 Quantitative‐PCR analysis (qPCR)

To narrow down the deletion breakpoints in each patient, we used real‐time qPCR to test the relative copy number of various strategically designed amplicons located along the *MECP2* gene. Primers were designed from the genomic clone NM_004992.3 using Primer3 program (primer sequences and annealing sites in Supplementary Data S1). Briefly, our qPCR strategy was based on generating standard curves for each *MECP2* amplicon and for the autosomal reference gene *MTHFR* (OMIM*607093). These standard curves defined the relationship between the input DNA concentration and the C_t_ value.

The real‐time qPCR was performed with the GoTaq Master Mix kit (Promega Corp., USA) for ABI 7500 Real‐Time PCR System (Applied Biosystems, Foster City, CA) and the PowerUp SYBR Green Master Mix kit for the QuantStudio 6 Flex Real‐Time PCR System (both from Applied Biosystems, USA). All reactions were conducted in triplicate with the average of each triplicate group used for quantitative analysis. Product specificity was assessed by melting curve analysis. The *MECP2* amplicon of interest and the *MTHFR* reference amplicon were amplified separately for each patient and for three normal female controls, yielding a copy number variant for each.

### 2.4 Long‐range PCR amplification and Sanger sequencing of deletion junctions

Once the deletions' breakpoints had been narrowed down to a sufficiently small region by qPCR, primer sites in the regions immediately flanking the breakpoints were selected for long‐range PCR amplification. As the precise size of the junction fragment in each patient was unknown, several different PCR conditions were tested and optimized. Long‐range PCR was performed with the Expand High Fidelity PCR System kit (Roche, Mannheim, Germany). This protocol was carried out in accordance with the manufacturer's instructions on a SimpliAmp Thermal cycler (Applied BioSystems, Waltham, MA). The PCR products were sequenced using a Big‐Dye® Terminator version 3.1 Cycle Sequencing Kit in an Applied Biosystems 3,730/DNA Analyzer (Applied BioSystems, Waltham, MA). The raw data were analyzed with Chromas trace viewer (http://technelysium.com.au/wp/chromas/). The sequences of the junction fragments were aligned to the reference sequence of *MECP2* (NM_004992.3) using Genomatix diAlign® program (local multiple alignment; http://www.genomatix.de/cgi-bin/dialign/dialign.pl).

### 2.5 X chromosome inactivation assay (XCI)

The XCI status of all 21 female patients was determined by the analysis of the methylation status of the highly polymorphic trinucleotide X‐linked androgen receptor (*AR*; OMIM*313700) locus. For each subject, 50 ng of genomic DNA was digested separately with *Hpa*II restriction enzyme (New England Biolabs, Beverly, MA) in accordance with the manufacturer's instructions. A region between 252 and 327 bp of the locus was PCR amplified from digested and undigested DNA using fluorochrome‐labeled primers. Samples were electrophoresed on an ABI Prism Genetic Analyzer 3130, and the peak areas were quantified using Gene Mapper v4.0 software (Applied Biosystems, Foster City, CA).

## 3 RESULTS

For a total of 21 patients without a point mutation or small *indel* detected in the *MECP2* coding region, MLPA was carried out, and at least one exonic probe was missing in each patient (see Supplementary Data S2). These patients were classified depending on the affected exons: only exon 4 of *MECP2* was affected in five patients (P1 to P5), exons 3 and 4 in nine patients (P6 to P15), exons 3, 4, and *IRAK1* gene in four patients (P16 to P19), exon 4 and *IRAK1* in one patient (P20), and exons 1 and 2 in one patient (P21) (see Table 1). All deletions have been confirmed to be de novo*.*

**Table 1:** All the collected data from the patients; genomic information is based on the GRCh38/hg38 and the accession number NM_004992.3 (NG_007107.2) for *MECP2* gene

Patient ID	Clinical phenotype	Clinical severity score	Age of scoring, yr.	*MECP2* exons deleted	Breakpoint	Deletion Size	ICX
P1	Classic	8	11	4	c.1153_2387del	1,235 bp	72:28
P2	Classic	NA	NA	4	c.1157_3664del	2,508 bp	88:22
P3	Classic	11	10	4	c.1041_4447del	3,407 bp	64:36
P4	Classic	10^a^	17	4	c.1164_4665del	3,502 bp	87:13
P5	Classic	15	2	4	c.1164_1461+3282del	3,580 bp	58:42
P6	Classic	NA	NA	3 and 4	c.27‐1125_1146del	3,001 bp	55:45
P7	Classic	NA	NA	3 and 4	c.27‐5834_1166del	7,720 bp	58:42
P8	Classic	NA	NA	3 and 4	c.27‐10677_1192del	12,599 bp	88:12
P9	Classic	NA	NA	3 and 4	c.(26+1_27‐1)_(378_1385)del	≈ 11.5 kb	82:18
P10	Classic	17	10	3 and 4	c.27‐7985_1209del	9,924 bp	51:49
P11	Classic	10	6	3 and 4	c.27‐2950_1170del	4,850 bp	62:38
P12	Classic	13^a^	6	3 and 4	c.27‐6312_1301delinsTG	8,343 bp	68:32
P13	Classic	14	4	3 and 4	c.(26+1_27‐1)_(989_1241)del	≈19.15 kb	74:26
P14	Classic	NA	NA	3 and 4	c.27−15131_1461 + 7939del;insGGATCAGGT	25,261 bp	76:24
P15	Classic	13	8	3 and 4	c.(26+1_27‐1)_(1461+4313_1461+5337)del	≈22.5 kb	83:17
P16	Classic	14	3	3, 4 and *IRAK1*	g.(154092184_154032557)_(154000172_153997133)del	≈ 50.7 kb	94:6
P17	Classic	10	10	3, 4 and *IRAK1*	g.(154092184_154032557)_(153986984_153969656)del	≈ 77.9 kb	88:12
P18	Classic	15	21	3, 4 and *IRAK1*	g.(154092184_154032557)_(154021813_154018990)del	≈ 28.3 kb	64:36
P19	Classic	15	16	3, 4 and *IRAK1*	c.[27‐16409_1201delinsGGGGGCC; 1202_1460+2170inv; 1460+2171_1460+12766delinsTCTGCACGGGG]	18339bp and 10596bp	97:03
P20	Classic	14^a^	NA	4 and *IRAK1*	g.154030942_154003453del	27,589 bp	74:26
P21	Classic	17	NA	1 and 2	g.(154128954_154097731)_(154092184_154032557)del	≈ 85 kb	73:27

Abbreviation: NA, not available.

a Indicates the lack of information for some of the clinical features (See Supplementary Data S4); so the given score is the result of the data we were given by the clinicians.

**Figure 1:**
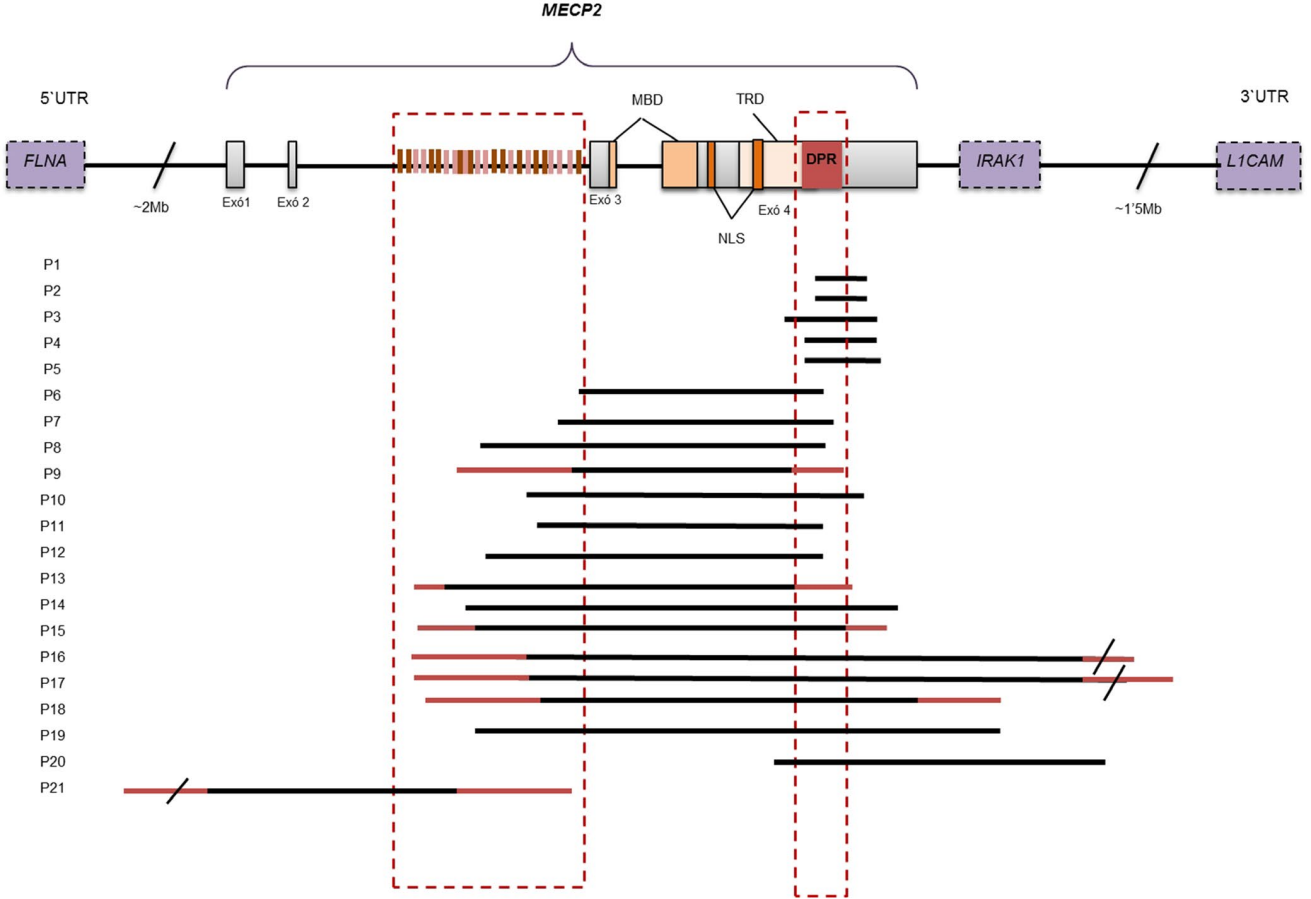
Position of the deletions in *MECP2.* Note that there are two regions prone to harbor a breakpoint. The black line indicates the region known with certainty to be deleted; the red line designates the region where only qPCR information is available

To validate the MLPA technique, quantitative‐PCR (qPCR) was performed in all patients with suspected deletions. qPCR analysis of the respective regions showed results compatible with deletion. Relative ratios of 0.5 ± 0.2 were suggestive of a deletion, whereas ratios of 1 ± 0.2 were indicative of a normal copy number for that region (for more information about the narrowing down of the deletion in each case, see Supplementary Data S3). Several PCR primer sets were evaluated to identify the ones that flank the deletion junction and could amplify, such that Sanger sequencing could be performed. For patients P1, P2, P3, P4, P5, P6, P7, P8, P10, P11, P12, P14, P19, and P20, different pairs of primers (Supplementary Data S1) successfully amplified the junction fragment that was subsequently sequenced (Figure 1). The deletions we have characterized range from 1,235 bp to 85 kb and involve different exons of *MECP2*, sometimes even ending in nearby genes. In case P19, we found a large inversion alongside the deletion.

With the patients' clinical information, we assigned each patient a severity score based on Pineda score. This scoring system gathers information about clinical features for classic RTT such as the patient's age of onset of the first sign, the presence of microcephaly, the ability to sit alone, ambulation, epilepsy, hand use, onset of stereotypies, respiratory function, and language. Complete information of approximately 12 patients was available; three more patients' reports lack one of those aspects, and no information was available about the other six girls. Even if each patient has a unique phenotype, there are some characteristics that are present more frequently among them: 60% of the girls present the first signs before the age of 12 months, 92.3% of the girls have microcephaly, 64.3% have respiratory problems, 93.3% suffers from epilepsy, 93.3% lost their hand use, 80% began with the stereotypies before 36 months of age and 60% before 24 month, and 86.6% lost their language (see Supplementary Data S4). No clear correlation was identified between the size of the deletion in base pairs and the severity of the phenotype, although there seems to be a trend when taking into account the deleted exons: patients with deletions comprising only one exon of *MECP2* have milder symptoms than those with deletions that involve both exons 3 and 4 or contain *IRAK1* as well.

All 21 patients were heterozygous at the *AR* locus and were thus informative for the assay. Patients P2, P4, P8, P9, P15, P16, P17, and P19 have skewed XCI (defined here as ≥80% activity of one X chromosome). However, 13 of 21 girls have an XCI of >70%; even if we do not consider these results completely skewed, they may indicate a cellular trend to inactivate the mutant allele. This phenomenon could account for why some of our patients do not have such a high score as we may have expected for the size of their deletions (for example P15 or P17). Unfortunately, we lack the clinical information about some of the girls with skewed X. The results for each subject are listed in Table 1 along with a summary of all other results obtained.

## 4 DISCUSSION

In this study, we have screened a cohort of 21 classic RTT patients with large *MECP2* deletions detected by MLPA. Subsequent qPCR analysis has confirmed the presence of large deletions in all of them. The deletion breakpoints were further characterized by qPCR and long‐range PCR with the aim of defining the precise endpoints at the nucleotide level. That last step was achieved in 14 out of 21 patients. The large number of GCs and all the repetitive sequences found in the intronic region of the gene and in the intergenic zones may have increased the difficulty for the polymerase to amplify our targeted products in those cases. Additionally, after characterizing the case of P19 in which an inversion occurred between two different deletions, we cannot dismiss the possibility that more complex rearrangements are present in the genome of those patients interfering with the correct hybridization of the primers. With the introduction of next generation sequencing and specifically, the whole genome sequencing (WGS), the delineation of those unresolved cases could be achieved; although WGS is still not affordable in order to use it as a routine technique for Rett Syndrome patient testing.

Our results showed a wide range of genotypes, from deletions affecting only a single exon to others involving almost the entire *MECP2* gene and the gene located downstream, *IRAK1*. We found only one patient with a deletion in exons 1 and 2 and part of the promoter region of the *MECP2* gene. This is in accordance with previous findings, although a small number of deletions have been reported affecting exons 1 and 2(Archer et al., 2006; Erlandson et al., 2003; Hardwick et al., 2007; Ravn et al., 2005).

Nine patients (P1, P2, P4, P5, P6, P7, P8, P11, and P12) whom we successfully characterized had a breakpoint in the “deletion‐prone region” (DPR, GRCh38/hg38 chrX:154,030,619‐154,030,770), as defined by Laccone et al. (2004)(Laccone et al., 2004). Another two patients (P3 and P10) had their breakpoint close to this region (less than 80 bp away). There are two patients (P9 and P13) who could have one of their breakpoints in the DPR as well, but since they are not fully characterized, we cannot confirm this conclusion (see Figure 1). Our finding together with previous studies (Hardwick et al., 2007; Laccone et al., 2004; Ravn et al., 2005; Schollen, Smeets, Deflem, Fryns, & Matthijs, 2003) could better define the junction sequence of the large MECP2 deletions, since 22 of 42 (52.3%) rearrenged alleles have the breakpoint in the DPR. This region is also the hotspot for the smaller deletions (<500 bp) confined within exon 4. The repetitive nature of the DPR has been considered the major cause of genomic instability there; these include the presence of direct and inverted small repeats, the abundance of polypurine residues in the antisense strand and the presence of the χ‐sequence GCTGGTGG, which has been found to be highly recombinogenic in the *Escherichia coli* genome (Stahl, Kobayashi, Stahl, & Huntington, 1983). It has been suggested that this sequence stimulates the recombinase BC‐dependent system and is responsible of certain deletions that cause human diseases (Amor, Parkert, Globerman, New, & White, 1988; Marshall, Isidro, & Boavida, 1996).

In addition to the DPR, eight patients whom we successfully characterized and seven in whom long PCR failed had a breakpoint in the same intron 2 region (GRCh38/hg38 version chrX:154033244‐154052415). The RepeatMasker program (http://www.repeatmasker.org) revealed that 48.9% of this intronic region consists on interspersed repeats, and 17.9% of them are Alu elements. It has been previously hypothesized that those abundant Alu elements interact with the χ‐sequence near the DPR making these types of large rearrangements in *MECP2* possible and recurrent (Laccone et al., 2004; Rüdiger, Gregersen, & Kielland‐brandt, 1995). Additionally, Alu has proven to be involved in other genomic rearrangements in different genes (Gu et al., 2015; López et al., 2015; Peixoto et al., 2013). The data we provide contribute to strengthen the theory that all these rearrangements do not occur randomly across the gene and its surroundings but in focal areas. Once the deletion is precisely delimited, studies to correct this mutation by CRISPR‐Cas9 technology could be considered to regain a complete and functional *MECP2,* among other strategies the cell possess such as homology repair*.*

**Figure 2:**
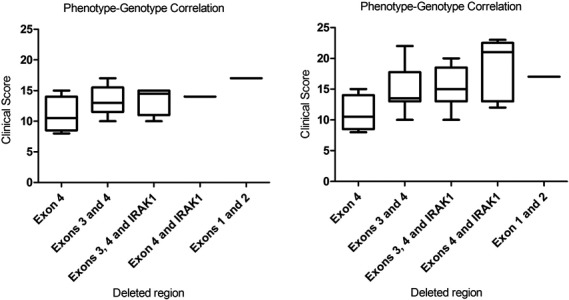
Phenotype–genotype correlation according to the deleted region. The left side corresponds to the correlation based on our patients. The right side shows the same correlation based on our patients and the ones reported by Hardwick et al. (2007)

We have attempted to establish genotype–phenotype correlations with our patient cohort. Although no clear correlation between the deleted exons and the clinical severity has emerged from this study, we can appreciate some trends (see Figure 2, left). The patient with the deletion involving exons 1 and 2 has a severe phenotype, which seems reasonable because those exons contain the starting sites for both isoforms of *MECP2* and that without that signal, no product could be generated a priori*.* Patients with a deletion in exon 4 show the mildest phenotype compared to the remaining combinations. This finding can be explained because none of our five deletions in exon 4 occurs in any of the main functional domains of the protein. An exception could be P5 who has the highest score of the group but, in this particular case, the patient was only 2 years old when the score was set, so it can still improve in the following years. Some authors have claimed that deletions involving *IRAK1* generate a more severe phenotype (Hardwick et al., 2007). *IRAK1* is the interleukin 1 receptor‐associated kinase and plays a critical role in initiating an innate immune response against foreign pathogens. In our cohort, little difference can be seen when *IRAK1* is added to the deletion but we must admit that the used checklist and scoring system does not take into account the severity or recurrence of the infections of the patients, features that may allow differentiating the effect of having or not *IRAK1* deleted. If we use to determine the severity of the phenotype the Pineda’s clinical score, patients reported by Harwick et al. (2007) and patients from our cohort trends to be similar (see Figure 2 right). However, we are aware that exceptions exist and that, sometimes, patients with the same or very similar deletion present a very different phenotype. For example, P4 and P5 have a similar deletion but their score differs by five points. Other examples are the cases described by Bebbington et al. (2012), Mittal, Kabra, Juyal, and BK (2011) or Erlandson et al. (2003). All these cases may suggest another mechanism that alters the direct effect caused by the deletions, possibly a specific methylation pattern causing another molecular alteration in another gene or regulatory domain. Such is the case of the brain‐derived neurotrophic factor*, BDNF* (OMIM*113505)*,* gene which is known to protect the carriers of the polymorphism p.Val66Met against early onset epilepsy (Li & Pozzo‐Miller, 2014).

Considering the molecular and clinical effect that the lack of a noteworthy part of the coding region of the gene can cause, we were expecting very severe phenotypes. However, the scores in our cohort were not always correlated. The XCI issue has frequently been considered in research on RTT as a potential explanation for the diverse phenotypes generated from the same genotype (Shahbazian, Sun, & Zoghbi, 2002). It has been shown that different cell types can have a different XCI pattern and that the one observed in blood lymphocytes may not be the same as in the brain, the organ in which the majority of symptoms of RTT occur (De Hoon, Monkhorst, Riegman, Laven, & Gribnau, 2015). This phenomenon has accounted for how some females carry a mutation in *MECP2* and are asymptomatic, because of the extreme inactivation of the X chromosome that harbors the aberrant allele (Shahbazian et al., 2002). A similar scenario occurs in mothers carrying a duplication of *MECP2,* who have a skewed XCI and are phenotypically normal unlike their affected offspring who develop *MECP2* duplication syndrome (Van Esch, 2011; Lim, Downs, Wong, Ellaway, & Leonard, 2017). Eight of our patients have a skewed XCI pattern, nine if we lower the threshold to <75% like other authors have done (Hardwick et al., 2007). However, if we lower it to <70% of XCI, four more girls can be included, making a total of 13 patients without a complete random pattern, which could suggest positive selection of cells with an inactivated copy of the defunct *MECP2* allele as a protective mechanism against such large deletions. This hypothesis could explain the relatively mild phenotypes of our cohort. Additionally, we could perform allele specific XCI in two of our patients, P8 and P20 (Personal Data). P8 presents an allele‐specific inactivation of 6:94, so most of the mutated allele was inactive, as we expected. Unfortunately, no clinical data were available for this patient. In the case of P20, this technique showed a random inactivation of the gene, so the score might not be so high because no functional domain is present in the deletion and, therefore, the molecular implications for the loss might not be as critical as if they were.

In conclusion, molecular characterization of large rearrangements in *MECP2* is possible in the majority of the cases using the methodology we have exposed. Analysis of that information supports the theory that the 3’ end of exon 4 and intron 2 are prone to suffer breaks that can lead to these deleterious big deletions. In addition, gathering clinical data enabled us to define a new set of features that are present in patients with large deletions, such as microcephaly, epilepsy, loss of hand use, loss of language, or onset of stereotypies before 36 months. These data will be very helpful for genetic counseling. A correlation between the severity of the patient and the position of the deletion shows that it is milder when only one exon is deleted and more severe when exons 3 and 4 and *IRAK1* are also involved. In addition, it seems that there is a cellular trend that inactivates the chromosome with the aberrant allele alleviating the final phenotype.

## CONFLICT OF INTEREST

The authors declare no conflict of interest.

## AUTHORS’ CONTRIBUTIONS

J.A. and M.P. conceived and supervised the study. S.V., A.P.A., M.R., E G., N.B., and P.P. performed the experiments and collected the data. J.A., S.V., A.P.A., M.R., E.G., N.B., and P.P. analyzed the results. J.A, M.P., and the Rett Working Group provided the patients' samples and clinical and genetic information. A.P.A., S.V., and J.A. wrote the manuscript. All the authors reviewed the article critically for intellectual content.

## RETT WORKING GROUP

Hospital Sant Joan de Déu (Barcelona): Maria del Mar O’Callaghan (mocallaghan@sjdhospitalbarcelona.org); Àngels Garcia‐Cazorla (agarcia@sjdhospitalbarcelona.org)

Hospital San Borja Arriaran, Santiago de Chile (Chile), Facultad de Medicina Universidad de Chile: Dra Monica Troncoso (monicatroncososch@gmail.com); Dr Guillermo Fariña (guillermofarina@gmail.com)

Hospital Clínico Universitario Virgen de la Arrixaca (Murcia): Dra María Rosario Domingo (mrosario.domingo@gmail.com); Dr. Salvador Ibañez (salibmi@hotmail.com)

Hospital Infantil Universitario Niño Jesús (Madrid): Dr. García Peñas (jgarciadelarape.1961@gmail.com); Dra López Marín (laural.marin@hotmail.com); Dr. González Gutierrez‐Solana (luisggsolana@hotmail.com)

Hospital Materno Infantil‐ Hospital Infanta Cristina (Badajoz): Dr. Enrique Galán Gomez (egalan@unex.es)

Hospital Universitario Fundación Alcorcón (Madrid): Miguel Ángel Martínez Granero (MAMartinezg@fhalcorcon.es)

Hospital Universitario de Girona Dr. Josep Trueta (Girona): Dra María Obon (mobon.girona.ics@gencat.cat)

Parc Taulí Hospital Universitari (Barcelona): Dra Neus Baena Díez (NBaena@tauli.cat)
