# Supplementary material for: Characterization of large deletions of the MECP2 gene in Rett syndrome patients by gene dosage analysis
Source: Mol Genet Genomic Med. 2019 Jun 17;7(8):e793. doi: 10.1002/mgg3.793 (PMC6687651; doi:10.1002/mgg3.793)
Supplement: Supplementary file 1 [file MGG3-7-e793-s001.docx]

| **Gene** | **Primer name** | **Sequence 5'-3'** | **Tm** | **Hybridization area** |
| --- | --- | --- | --- | --- |
| ***MECP2*** | 60.1.1F | TTTATCTTAGTGGCCTCTTCGTG | 58,9 | Intron 2 (around 20kb from exon 3) |
|  | 60.1.1R | AACTACAACTCTTGCTGCGAACT | 58,9 |  |
|  | 60.1.2F | TAGGTCTACAGGCATGTTCAGGT | 60,6 | Intron 2 (around 20kb from exon 2) |
|  | 60.1.2R | TGTAATAATGGTTTCAGGCAAGG | 57,1 |  |
|  | 60.2.1F | TTCAAGCTGAACCTCATTCTCTC | 58,9 | Intron 2 (around 10kb from exon 3) |
|  | 60.2.1R | CTACAGCTCCAGGTGAACAGTCT | 62,4 |  |
|  | 60.3.1(2)F | GCTCTTGTTTAGTCCCTTTGGAT | 58,9 | Intron 2 (around 5kb from exon 3) |
|  | 60.3.1(2)R | ACTAATGGGAAAGATCTGGCTTC | 58,9 |  |
|  | 60.3.1(1)R | CATGCCTGTAATCCCAGTTACTC | 60,6 | Intron 2 (around 5kb from exon 3) |
|  | 60.3.2F | TGTGTTCAGATTTCGTCTTCTCA | 57,1 | Intron 2 (around 10kb from exon 3) |
|  | 60.3.2R | ACCTGAAACAGCAACGACTACAT | 58,9 |  |
|  | 60.4.1F | AATTACACCCCTTTCCTTTGTGT | 57,1 | Intron 2 (around 5kb from exon 3) |
|  | 60.4.1R | TAGAATAGGCATCTGAGGACAGC | 60,6 |  |
|  | M.e2-3.1R | TCCTCAGAAAAACCACCTATTCA | 57,1 | Intron 2 (around 11kb from exon 3) |
|  | M.e2-3.1F | CGGACTACTGATATGTCCCAAAC | 60,6 | Intron 2 (around 7,4kb from exon 3) |
|  | M.e2-3.2F | GAGCTTTCAGTGTAACCATCACC | 60,6 | Intron 2 (around 5,5kb from exon 3) |
|  | M.e2-3.3F | TGAATAGGTGGTTTTTCTGAGGA | 57,1 | Intron 2 (around 6,2kb from exon 3) |
|  | M.e2-3.4F | GATTGTGTCATCCCTTCAGTCTC | 60,6 | Intron 2 (around 7,9kb from exon 3) |
|  | M.e2-3.5F | ATACCTTTCATCTGCCTTGTTCA | 57,1 | Intron 2 (around 9,4kb from exon 3) |
|  | M.e2-3.6F | CCCTCAGTCTTTTCCTTCTCAAT | 58,9 | Intron 2 (around 3,8kb from exon 3) |
|  | M.e2-3.7F | TTCTAAGTGAGAACGTACGGTATTTG | 58,7 | Intron 2 (around 5,1kb from exon 3) |
|  | M.e2-3.8F | ACATGATCCCTCCTTGCCTATT | 58,4 | Intron 2 (around 15kb from exon 3) |
|  | 60.3.1_F | CTGGGCTAAGGGTAAACTCATTT | 59,9 | Intron 2 (around 5,1kb from exon 3) |
|  | 60.3.1_R | CAGCTCAGACACAGAAAGTCAAA | 59,7 |  |
|  | 60.4.2_F | CTGCAAATGCCAGATGCTTA | 60 | Intron 2 (around 12kb from exon 3) |
|  | 60.4.2_R | CCTTGTGGGCCAGACAGTAT | 60 |  |
|  | 60.5.3_F | ACTCACTGCAGCCTCAACCT | 60,1 | Intron 2 (around 15kb from exon 3) |
|  | 60.5.1_F | CCATCACCACATGCATCTTC | 59,9 | Intron 2 (around 15kb from exon 3) |
|  | 60.5.4_F | AGGCCCTGCATTTTCATTTT | 60,8 | Intron 2 (around 15kb from exon 3) |
|  | 1.1_F | CCAAACCCCTGGGATTAGAT | 60 | Intron 2 (around 19,8kb from exon 3) |
|  | 1.1_R | TCAGGGTCACCAGGAAGAAC | 60,1 | Exon 4 non coding sequence |
|  | M.4.6_F | GACCGTACTCCCCATCAAGA | 59,8 | Exon 4 coding sequence |
|  | M.4.6_R | GGAGACAAAGCAGGCCTATG | 59,9 | Exon 4 non coding sequence |
|  | M.4.5_F | AACAGAGAGGAGCCTGTGGA | 60 | Exon 4 coding sequence |
|  | M.4.5_R | AGTGAGAGGCCAGCTCAGAA | 60,3 | Exon 4 non coding sequence |
|  | M.4.4_F | AGGCAGGAGAGACAGTTGGA | 60 | Exon 4 non coding sequence |
|  | M.4.4_R | ATCCCGTCAGAGCAGAGAGA | 60,1 |  |
|  | M.4.2_F | CTTTCCCTTCCTCCATCCTC | 60 | Exon 4 non coding sequence |
|  | M.4.2_R | GGTGTCCCCTGTGTCTTTTG | 60,4 |  |
|  | M.4.0,7_F | AGCTGTGGTGTAGGGGCTTA | 59,8 | Exon 4 non coding sequence |
|  | M.4.0,7_R | GTCCTTTGGTAGTGCCAGGA | 60,1 |  |
|  | 61.1_F | GCTCCTTTTCCAGAGCATTC | 59 | Exon 4 non coding sequence |
|  | 61.1_R | CCCACTCCATGGTAGGAACT | 58,9 |  |
|  | 6.1.1F | AGTGCTGACTGTAGTGACCATTG | 60,6 | At the end of exon 4 |
|  | 6.1.1R | GTCCCAAAACACAAAAACTGAAG | 57,1 |  |
|  | 4B_R | CCAACTACTCCCACCCTGAA | 60 | Exon 4 non coding sequence |
|  | M.e3.75R | TAAGAAAGAAGAGAAAGAGGGCAA | 57,6 | 75bp before exon 3 |
|  | M.e4.24R | CTGCCTTTATTCTTGTTGGTTTG | 57,1 | 24bp before exon 4 |
|  | M.4.0,2_R | CCAGGTAGTTCTCAACACTGTCAC | 60 | Exon 4 non coding sequence |
|  | 150.1_R | GCCTCTGTGCTTTCCTCAAC | 60,1 | Exon 4 non coding sequence |
| ***IRAK1*** | IRAK1.R | CTGAATTCCTTCCTCCTTGTCTC | 60,6 | Around 1kb before *IRAK1* |
|  | IRAK1.2R | TAGAAGAAGGGGCTGGAGATG | 59,8 | Around 0,7kb before *IRAK1* |
|  | IRAK1.3R | GTCAAGGTGGGCTCTTCTTTG | 59,8 | Around 0,5kb before *IRAK1* |
|  | 153.1_F | CGACCCCCTTCACATAGAGA | 60,1 | Intron 9 of *IRAK1* |
|  | 153.1_R | CATCACAGGGAGTGCTGAGA | 60 |  |
|  | 153.3_F | ATGGGCCCTACTGATGAGC | 60 | Intron 8 of *IRAK1* |
|  | 153.3_R | TGGAACTTGGGGAGAGAAGA | 59,8 |  |
|  | 152.1_R | GTCCTTGAAGGCATTCTTCC | 58,7 | Intron 7 of *IRAK1* |
|  | 152.2_R | CCAGGTGTCAGGAGTGCTTT | 60,3 | Intron 7 of *IRAK1* |
|  | 152.3_R | CCACCAGTGCCACCTACC | 60 | Intron 5 of *IRAK1* |
| ***OPNLW*** | 220.1.OLW.F | ATTCATCCTCACAGAGTGTCCAG | 60,6 | At the beginning of *OPNLW* gene |
|  | 220.1.OLW.R | CCCAGCCGACTGTATCTCTAAAT | 60,6 |  |
| ***Between FLNA i MECP2*** | 220.1.1F | ATCCTCCTTTCTCATGTGGTTCT | 58,9 | At around 44kb from *MECP2* |
|  | 220.1.1R | CAAGGCAAGGCTGTGAACTC | 59,4 |  |
|  | 220.1.4F | GGCTAAGAAAACATGAGCAACAC | 58,9 | At around 44kb from *FLNA* |
|  | 220.1.4R | TCTGTGTCTCCGCTAAGAAGACT | 60,6 |  |
|  | 220.2.1F | AAGATAACTGGAAGTCCCCAAATAC | 59,7 | At around 15kb from *MECP2* |
|  | 220.2.1R | GCCTGCCATAATTTTTATTATTCCT | 56,4 |  |
|  | 220.2.2F | CTTTTAGATTGAACTGCTCCCATTA | 58,4 | At around 30kb from *MECP2* |
|  | 220.2.2R | GATCCCAAAGGTTAAGGATAAAGAA | 58,1 |  |
|  | 220.3.1F | CTGGTCTCCAACTCTAGCTTCAA | 60,6 | At around 33kb from *MECP2* |
|  | 220.3.1R | CTTCTCCAAGATCAGTCCTCAAA | 58,9 |  |
|  | 220.4.1F | CTGCACCTACCCTCAACTCTTTA | 60,6 | At around 35kb from *MECP2* |
|  | 220.4.1R | TAGGACATGAAATGGTGTGTCAA | 57,1 |  |
| ***Between IRAK1 i L1CAM*** | 154.1.1F | TAGCAGTTTGACTTGGCTGAAAG | 58,9 | Around 38kb from *IRAK1* |
|  | 154.1.1R | GGTATATCCCTGGTCCCAACTAC | 62,4 |  |
|  | 154.1.2F | ACAAGTTCCTATTGTTGCCCTTC | 58,9 | Around 76kb from *IRAK1* |
|  | 154.1.2R | AATTCTTTCTTGGGCAAGATCC | 56,5 |  |
|  | 154.1.3F | TAGTGTCTGGGCAGAAGAGGAC | 62,1 | Around 114kb from *IRAK1* |
|  | 154.1.3R | CTTTAAATGCCTTTTCCACCTG | 56,5 |  |
|  | 154.2.1(1)F | GTCAGCAAATATGGGTCAGTGAT | 58,9 | Around 91kb from *IRAK1* |
|  | 154.2.1(1)R | GAATCCATGTCTGCCTGAATTAC | 58,9 |  |
|  | 154.2.2(1)F | ACATGCTGATCTTTCTTCAGCTC | 58,9 | Around 104kb from *IRAK1* |
|  | 154.2.2(1)R | TCTTTCTTGCTTCCCCTTATTTC | 57,1 |  |
|  | 154.3.1F | TAGCACAGACAGTACGGAGAACA | 60,6 | Around 22kb from *IRAK1* |
|  | 154.3.1R | CACCTATTTTGTTGCAAATGACA | 60,6 |  |
|  | 154.3.2F | TCACAATGAAATCTCATGTCACC | 57,1 | Around 32kb from *IRAK1* |
|  | 154.3.2R | ACACTATCATGCATTCGCTTTTC | 57,1 |  |
|  | 154.3.3F | CGTTTACTTCTGCAGCATTTCTT | 57,1 | Around 40kb from *IRAK1* |
|  | 154.3.3R | AGGCTTTCTGCCCTAGAGACTAA | 60,6 |  |
|  | 154.3.4F | CAACACAGAAACTTCTGACATGC | 58,9 | Around 57kb from *IRAK1* |
|  | 154.3.4R | ACTGGTCTACTTCGGACTGTGTG | 62,4 |  |
|  | 154.4.1F | TCAGTGAATGAACAACTCCCTCT | 58,9 | Around 6kb from *IRAK1* |
|  | 154.4.1R | CGTACGTGTCTGTACTGTTTGGA | 60,6 |  |
|  | 154.4.2F | GAGTCTCCACACATTTCTTGCTT | 58,9 | Around 17kb from *IRAK1* |
|  | 154.4.2R | AGAATGGCCTCGTTCTTTTCTAC | 58,9 |  |
|  | 154.5.1F | AAACAAAAACAGACAAATGGGAGTA | 56,4 | Around 35kb from *IRAK1* |
|  | 154.5.1R | GAAATAGCAAACAGGACTGGTAAAA | 58,1 |  |
|  | 154.6.1F | CTGCGTCTCTGGCTAATTAAACTAA | 59,7 | Around 10kb from *IRAK1* |
|  | 154.6.1R | CTCTTTGCAGTGAGTCCAATAAACT | 59,7 |  |
|  | 154.6.2F | GCATCAGGCCTTAGTTTTCTTATTT | 58,1 | Around 37kb from *IRAK1* |
|  | 154.6.2R | TGAAGTTACTGAAGTTATGCGTCCT | 59,7 |  |
|  | 154.2.1_F | CTCCCTAGTCTCATGACCTTCAG | 59,4 | Around 49Kb before *L1CAM* |
|  | 154.2.1_R | TGTAGATAAGCCCAATGAGCAGT | 60,2 |  |
|  | 153.2_F | CACCAAGCCCAGCTAATGTT | 60,1 | Around 2Kb from *IRAK1* |
|  | 153.2_R | GAACGGAAGCTACCAGGACA | 60,3 |  |
| ***MTHFR*** | MTH_F | CCAAGGCCACCCCGAAGC | 68,4 | Exon 4 of *MTHFR* |
|  | MTH_R | AGGACGGTGCGGTGAGAGTG | 66,1 |  |

Supplementary Data 1: List of all the designed primers for both the qPCR and the long-PCR experiments.
