# Supplementary material for: Characterization of large deletions of the MECP2 gene in Rett syndrome patients by gene dosage analysis
Source: Mol Genet Genomic Med. 2019 Jun 17;7(8):e793. doi: 10.1002/mgg3.793 (PMC6687651; doi:10.1002/mgg3.793)
Supplement: Supplementary file 2 [file MGG3-7-e793-s002.docx]

| Patients | Nº Missing Probes | Affected exons | Missing probes | Used probemix |
| --- | --- | --- | --- | --- |
| 1 to 5 | 1 | 4 | P01768-L01332 | probemix P015-E1 |
| 6 to 12 | 6 | 3 and 4 | P10839-L11496, P10840-L11497, P01348-L12499, P10841-L11498, P10842-L12494 and P01347-L12498 | probemix P015-D1 |
| 13 | 6 | 3 and 4 | P10839-L23618, P01348-L24159, P14737-L24161, P10841-L24158, P18441-L12494 and P01347-L24157 | probemix P015-F1 |
| 14 | 11 | 3 and 4 | P10839-L23618, P01348-L24159, P14737-L24161, P10841-L24158, P18441-L12494, P01347-L24157, P18442-L24243, P01768-L13824, P18444-L24151, P18446-L23620 and P01769-L23834 | probemix P015-F1 |
| 15 | 10 | 3 and 4 | P10839-L23618, P01348-L24159, P14737-L24161, P10841-L24158, P18441-L12494, P01347-L24157, P18442-L24243, P01768-L13824, P18444-L24151 and P18446-L23620 | probemix P015-F1 |
| 16 to 17 | 9 | 3, 4 and *IRAK1* gene | P10839-L11496, P01348-L12499, P10841-L11498, P10842-L12494, P01347-L12498, P01768-L01332, P01769-L01333, P01770-L01334 and P10835-L12500 | probemix P015-E1 |
| 18 | 7 | 3, 4 and *IRAK1* gene | P10839-L11496, P01348-L12499, P10841-L11498, P10842-L12494, P01347-L12498, P01768-L01332 and P01769-L01333 | probemix P015-E1 |
| 19 | 8 | 3, 4 and IRAK1 gene | P10839-L11496, P01348-L12499, P10841-L11498, P10842-L12494, P01347-L12498, P01769-L01333, P01770-L01334 and P10835-L12500 | probemix P015-E1 |
| 20 | 5 | 4 and *IRAK1* gene | P01347-L12498, P01768-L01332, P01769-L01333, P01770-L1334 and P10835-L12500 | probemix P015-E1 |
| 21 | 6 | 1 and 2 | P03770-L13387, P02002-L01335, P03409-L02797, P10836-L11493, P03768-L03229 and P01349-L12497 | probemix P015-E1 |

Supplementary Data 2: List of the missing probes for each patient and the MLPA kits used.
