# Supplementary material for: Characterization of large deletions of the MECP2 gene in Rett syndrome patients by gene dosage analysis
Source: Mol Genet Genomic Med. 2019 Jun 17;7(8):e793. doi: 10.1002/mgg3.793 (PMC6687651; doi:10.1002/mgg3.793)
Supplement: Supplementary file 3 [file MGG3-7-e793-s003.pptx]

## Slide 1
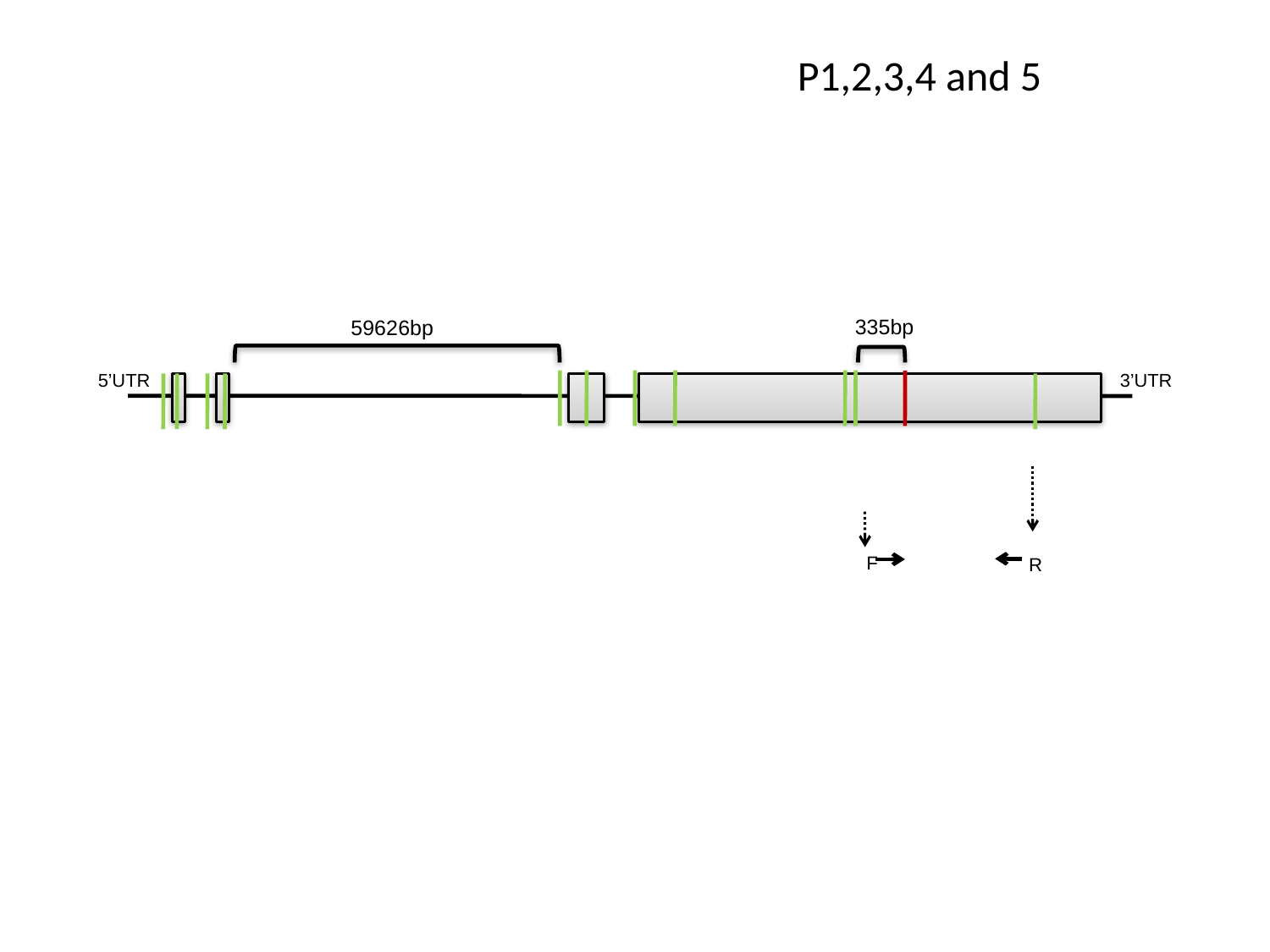

P1,2,3,4 and 5
335bp
59626bp
5’UTR
3’UTR
F
R

## Slide 2
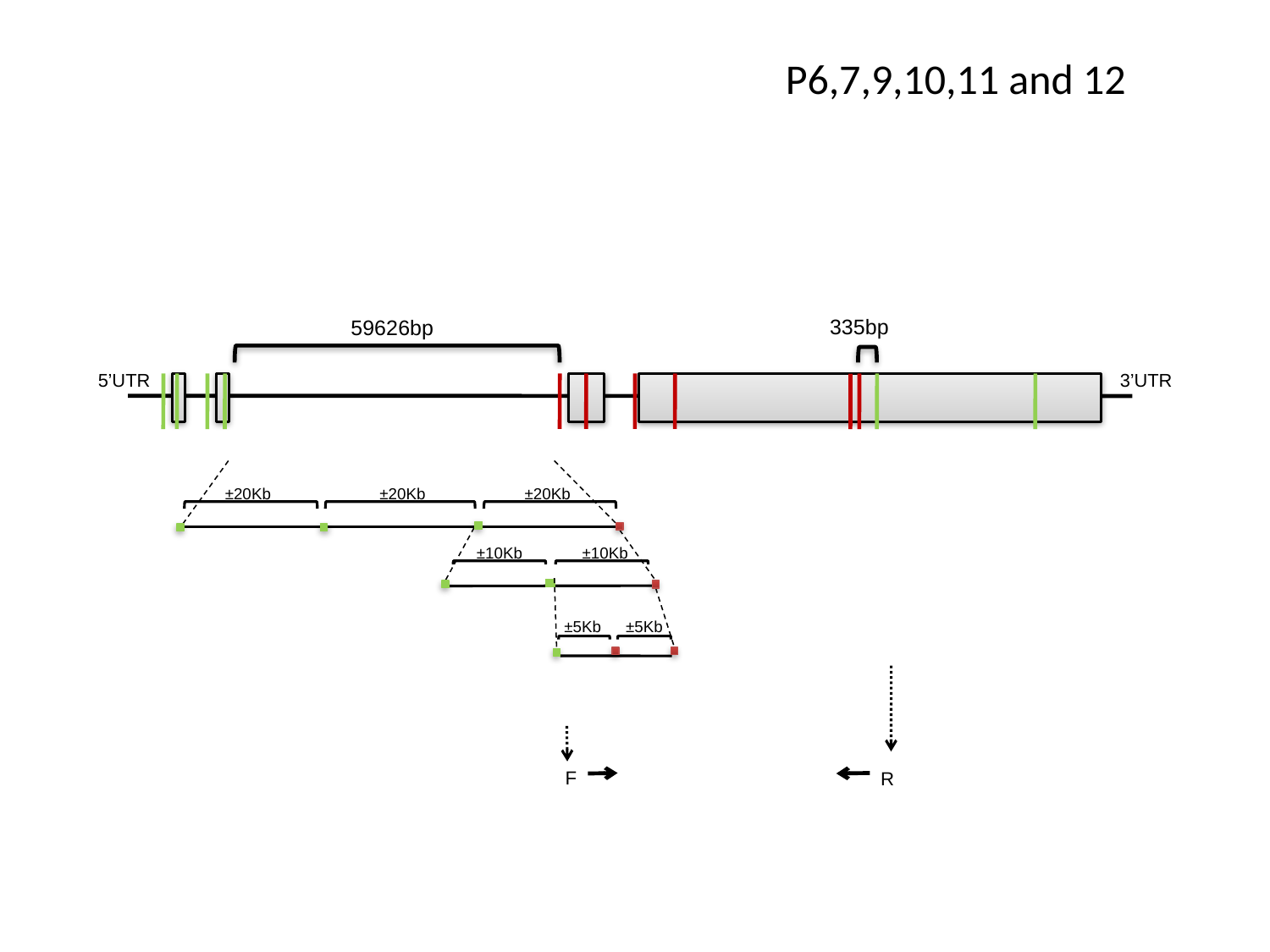

P6,7,9,10,11 and 12
335bp
59626bp
5’UTR
3’UTR
±20Kb
±20Kb
±20Kb
±10Kb
±10Kb
±5Kb
±5Kb
F
R

## Slide 3
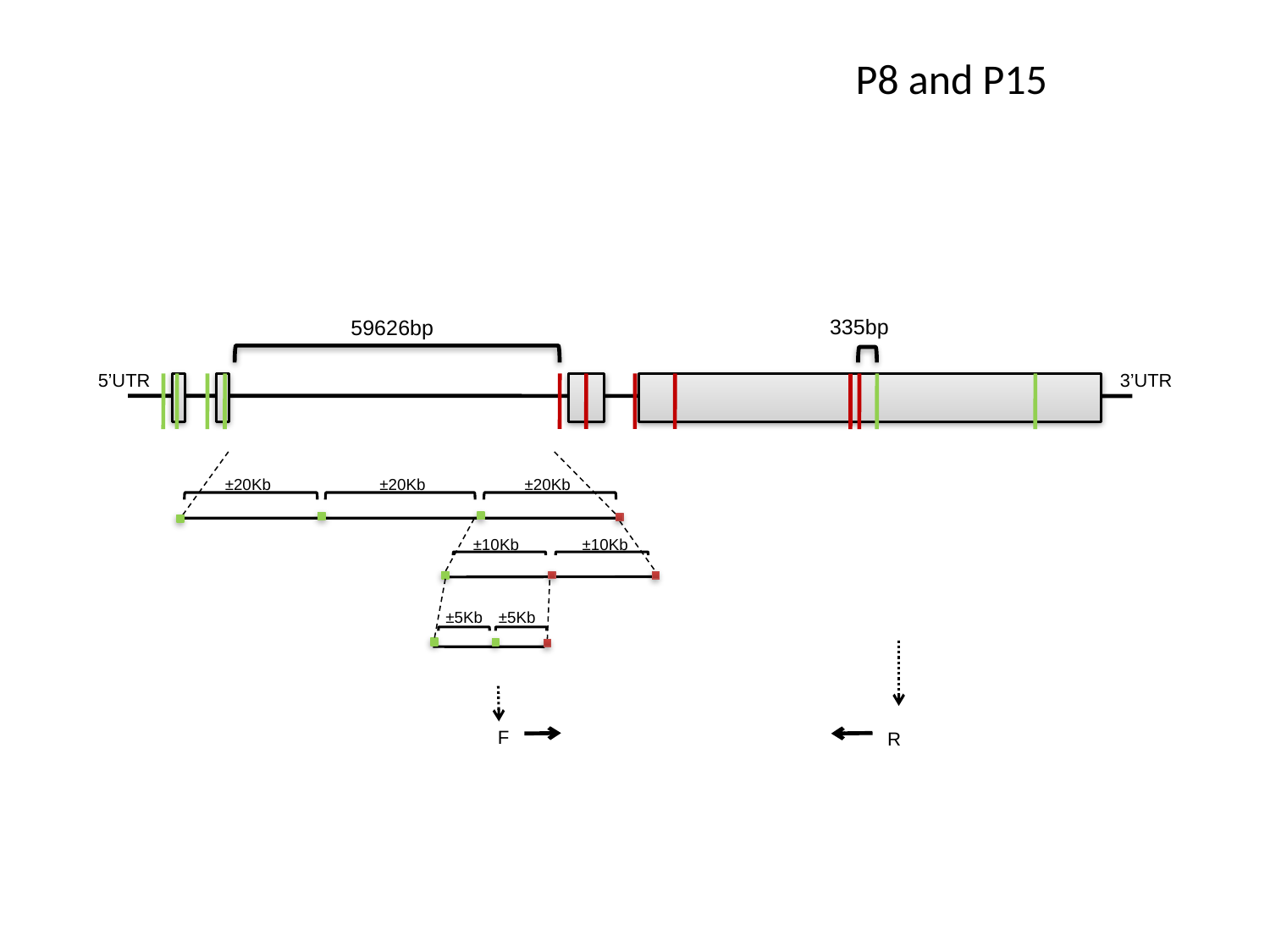

P8 and P15
335bp
59626bp
5’UTR
3’UTR
±20Kb
±20Kb
±20Kb
±10Kb
±10Kb
±5Kb
±5Kb
F
R

## Slide 4
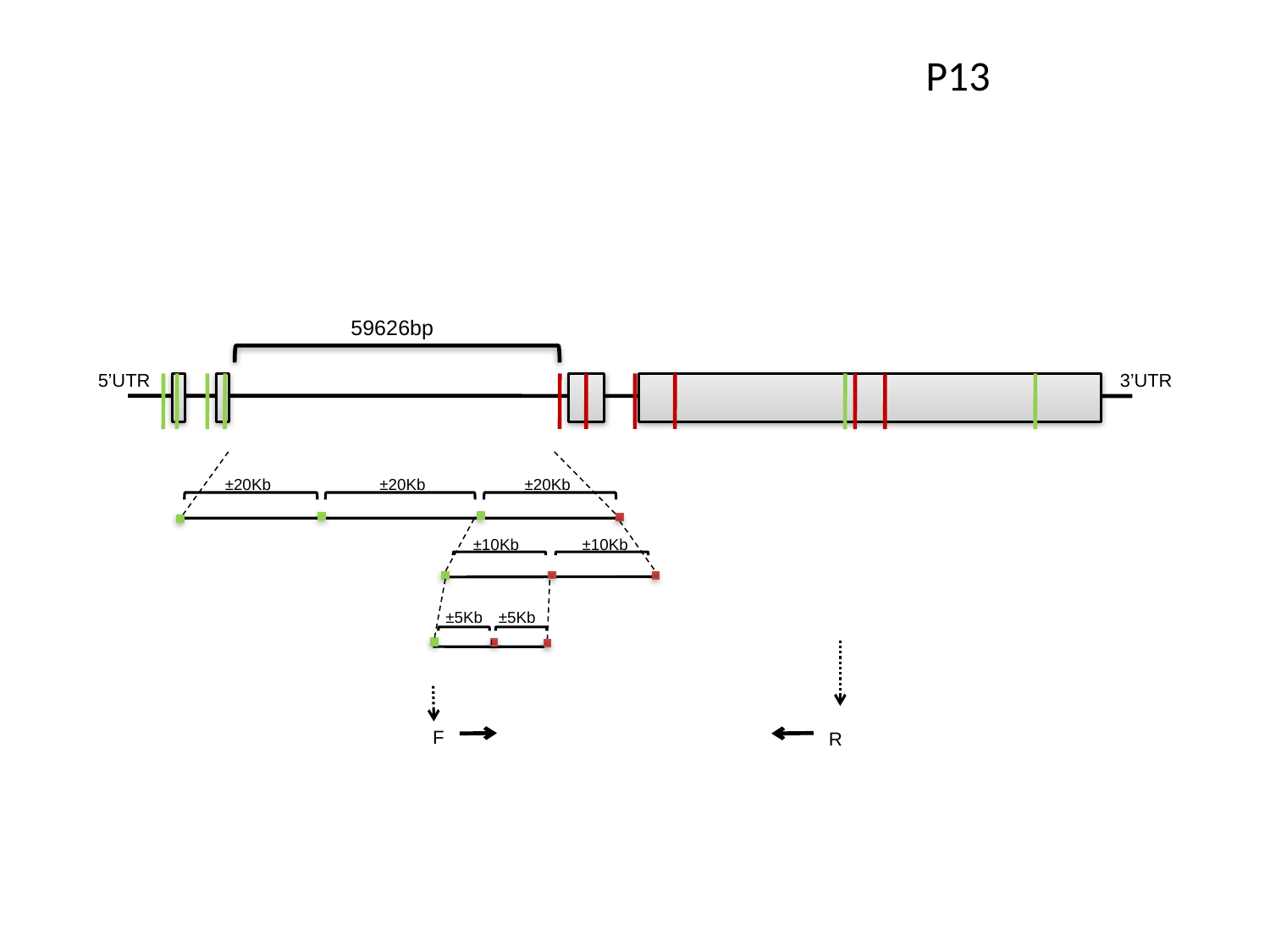

P13
59626bp
5’UTR
3’UTR
±20Kb
±20Kb
±20Kb
±10Kb
±10Kb
±5Kb
±5Kb
F
R

## Slide 5
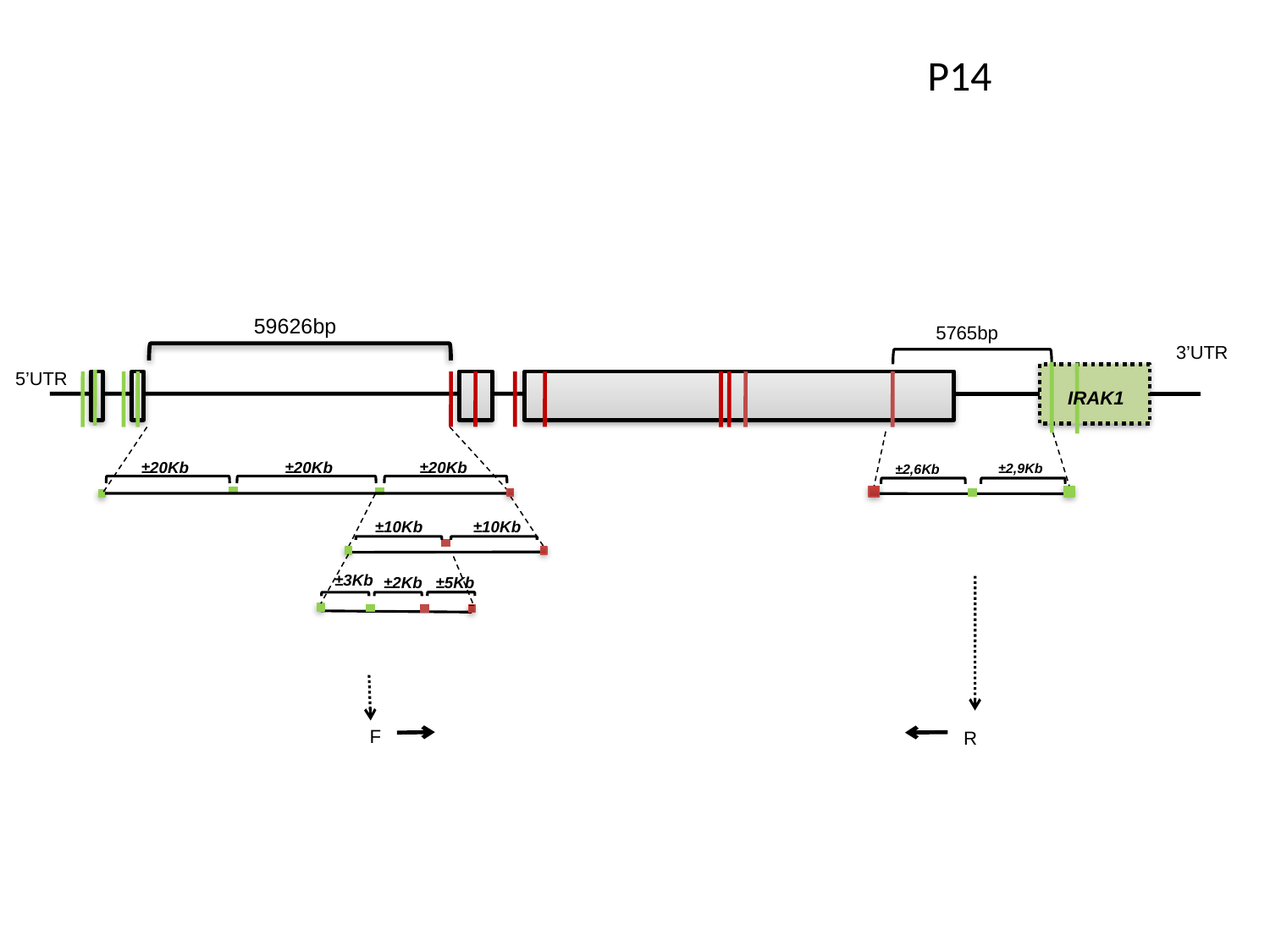

P14
59626bp
3’UTR
5’UTR
5765bp
IRAK1
±20Kb
±20Kb
±20Kb
±2,9Kb
±2,6Kb
±10Kb
±10Kb
±5Kb
±3Kb
±2Kb
F
R

## Slide 6
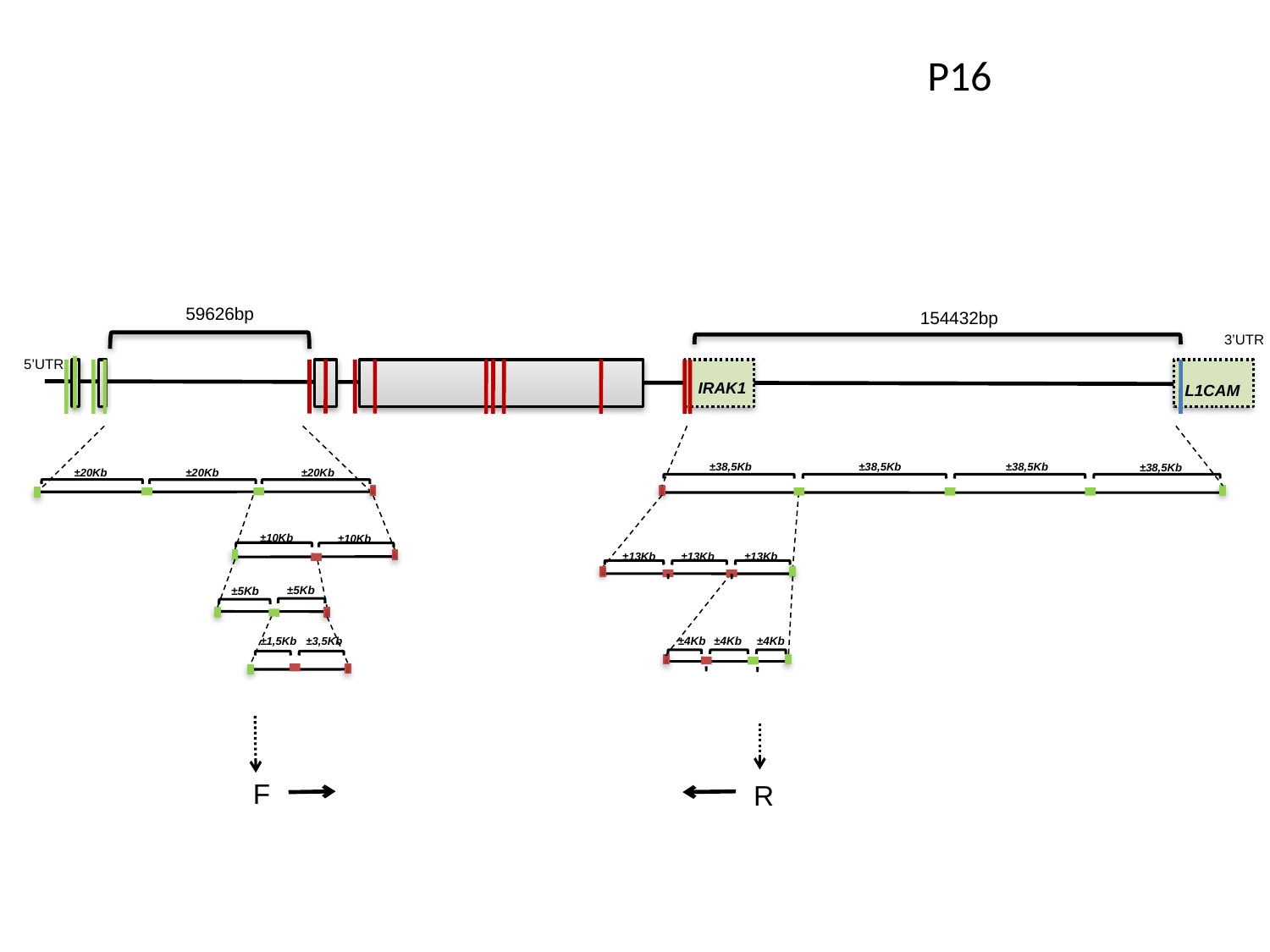

P16
59626bp
154432bp
3’UTR
IRAK1
L1CAM
5’UTR
±38,5Kb
±38,5Kb
±38,5Kb
±38,5Kb
±20Kb
±20Kb
±20Kb
±10Kb
±10Kb
±13Kb
±13Kb
±13Kb
'
'
±5Kb
±5Kb
±1,5Kb
±3,5Kb
±4Kb
±4Kb
±4Kb
 '
'
F
R

## Slide 7
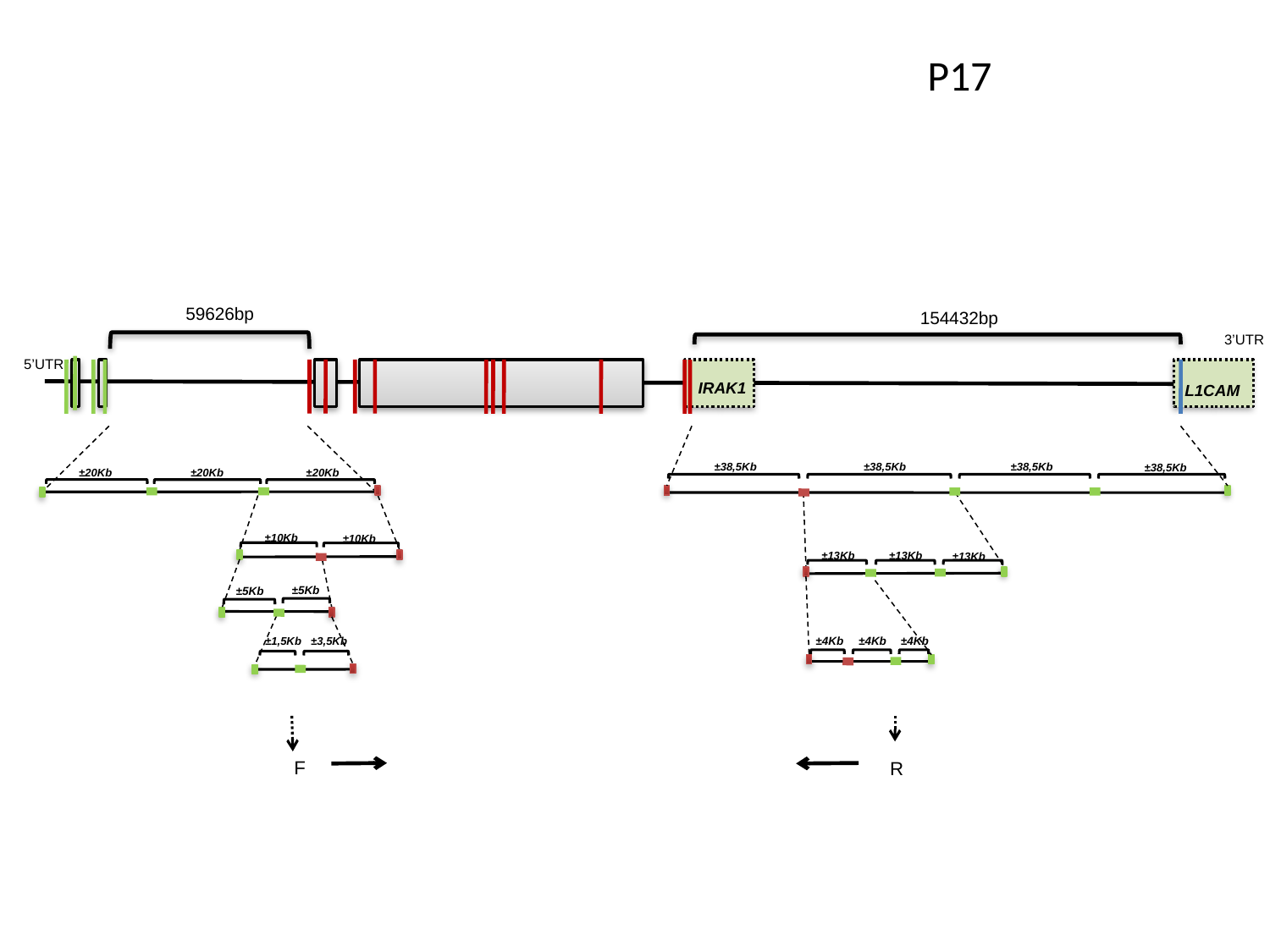

P17
59626bp
154432bp
3’UTR
IRAK1
L1CAM
5’UTR
±38,5Kb
±38,5Kb
±38,5Kb
±38,5Kb
±20Kb
±20Kb
±20Kb
±10Kb
±10Kb
±13Kb
±13Kb
±13Kb
±5Kb
±5Kb
±1,5Kb
±3,5Kb
±4Kb
±4Kb
±4Kb
F
R

## Slide 8
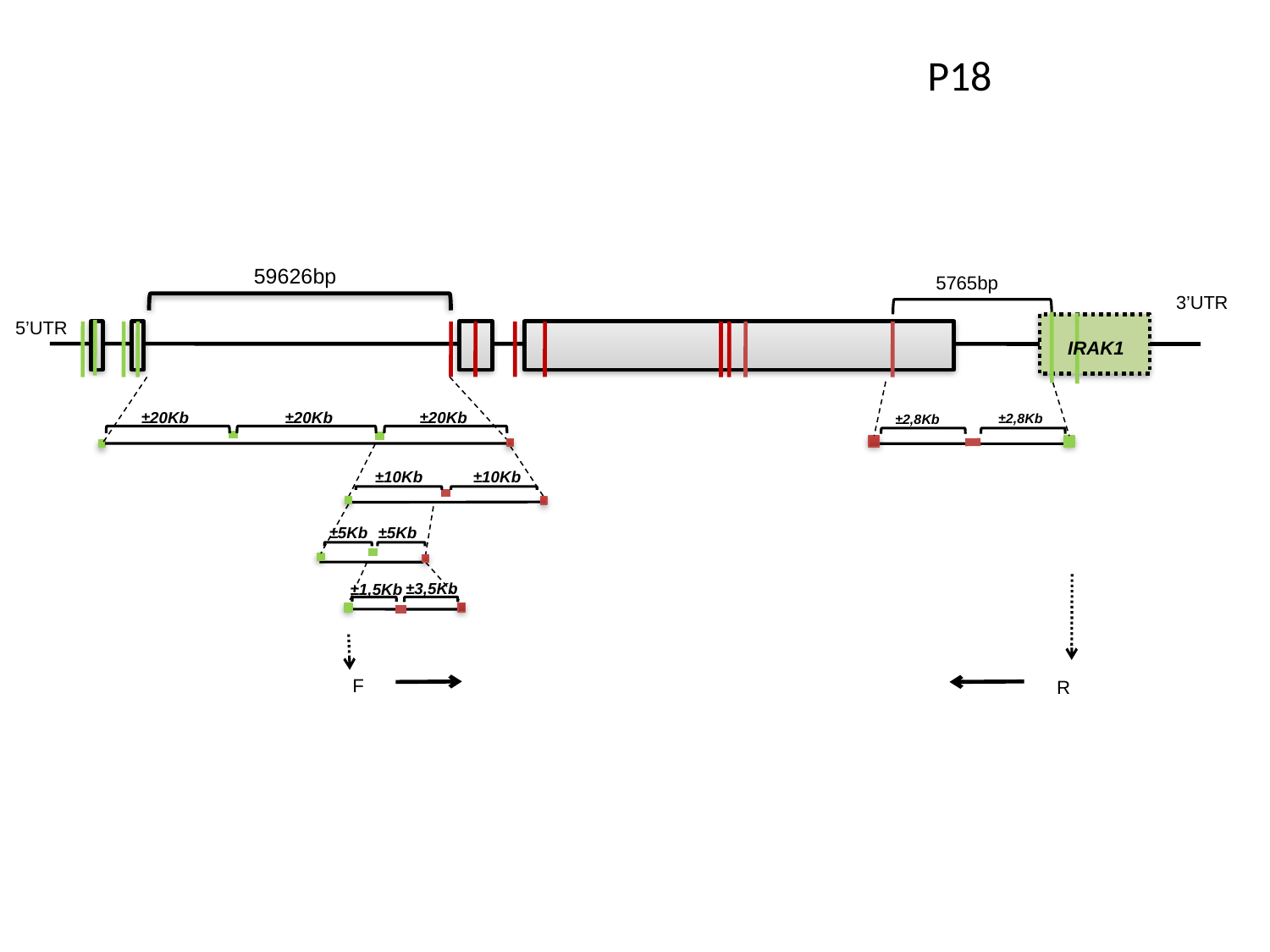

P18
59626bp
3’UTR
5’UTR
5765bp
IRAK1
±20Kb
±20Kb
±20Kb
±2,8Kb
±2,8Kb
±10Kb
±10Kb
±5Kb
±5Kb
±3,5Kb
±1,5Kb
F
R

## Slide 9
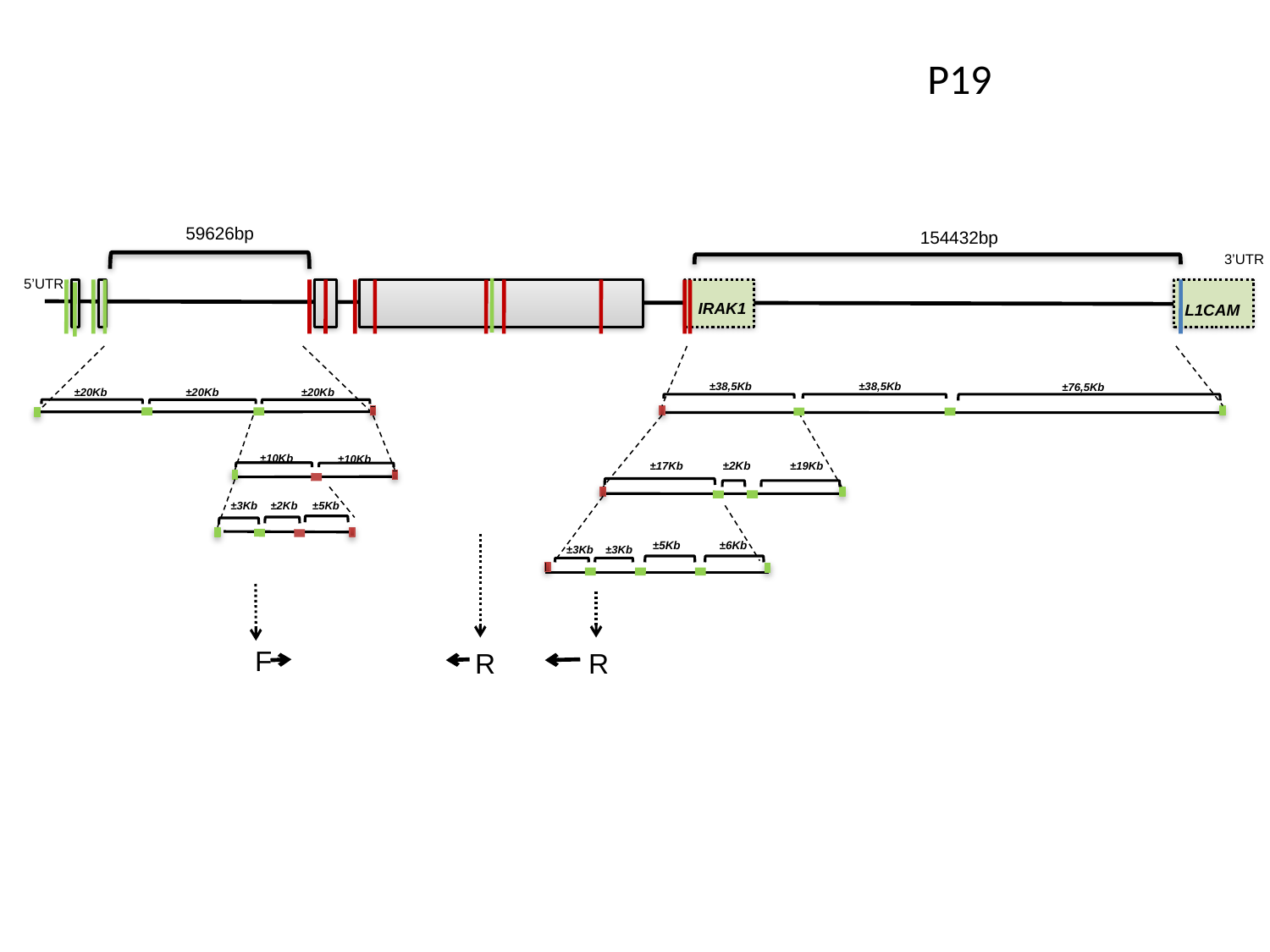

P19
59626bp
154432bp
3’UTR
IRAK1
L1CAM
5’UTR
±38,5Kb
±38,5Kb
±76,5Kb
±20Kb
±20Kb
±20Kb
±10Kb
±10Kb
±17Kb
±2Kb
±19Kb
±3Kb
±5Kb
±6Kb
±3Kb
±2Kb
±5Kb
F
R
±3Kb
R

## Slide 10
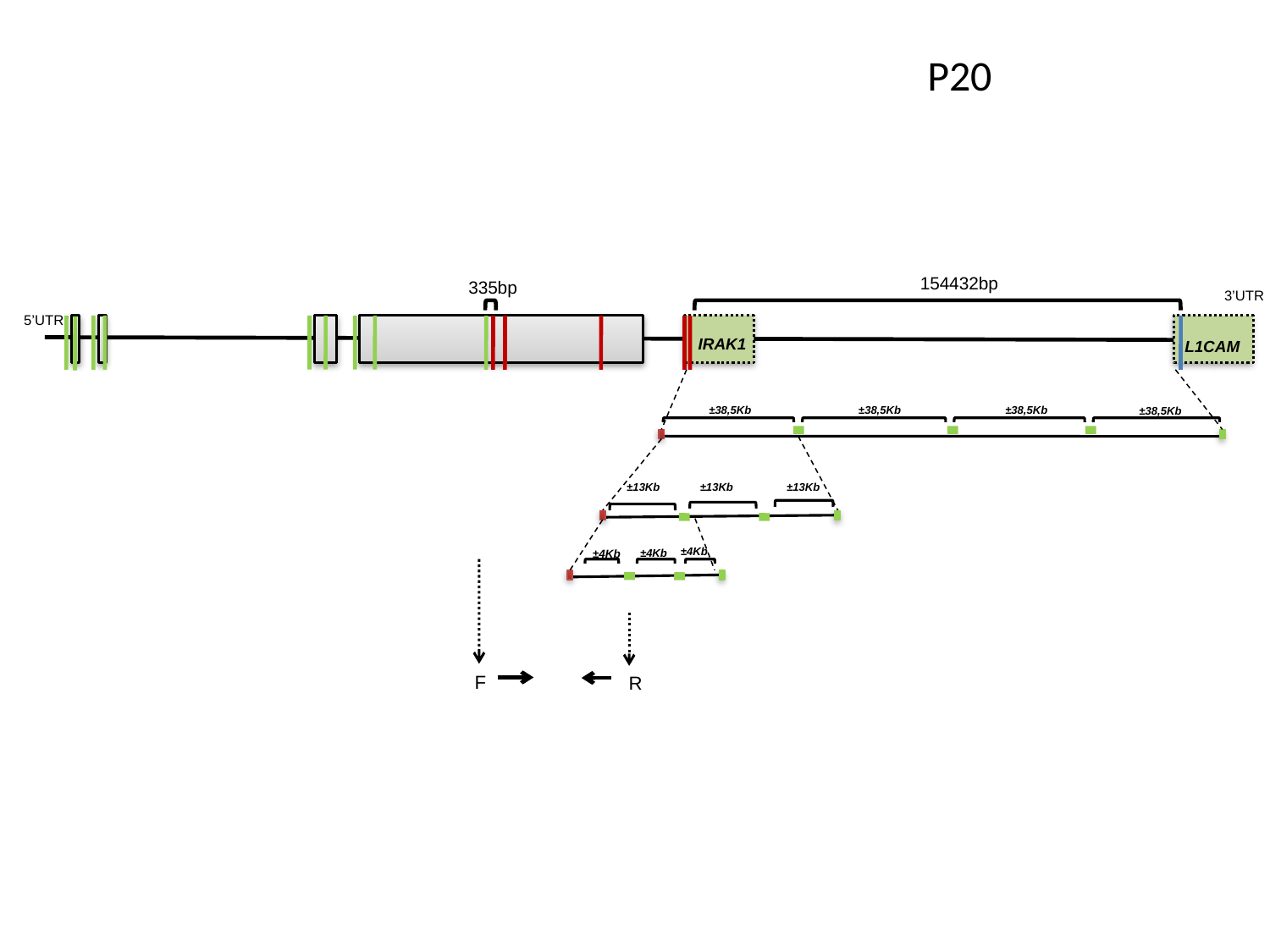

P20
154432bp
335bp
3’UTR
5’UTR
IRAK1
L1CAM
±38,5Kb
±38,5Kb
±38,5Kb
±38,5Kb
±13Kb
±13Kb
±13Kb
±4Kb
±4Kb
±4Kb
F
R

## Slide 11
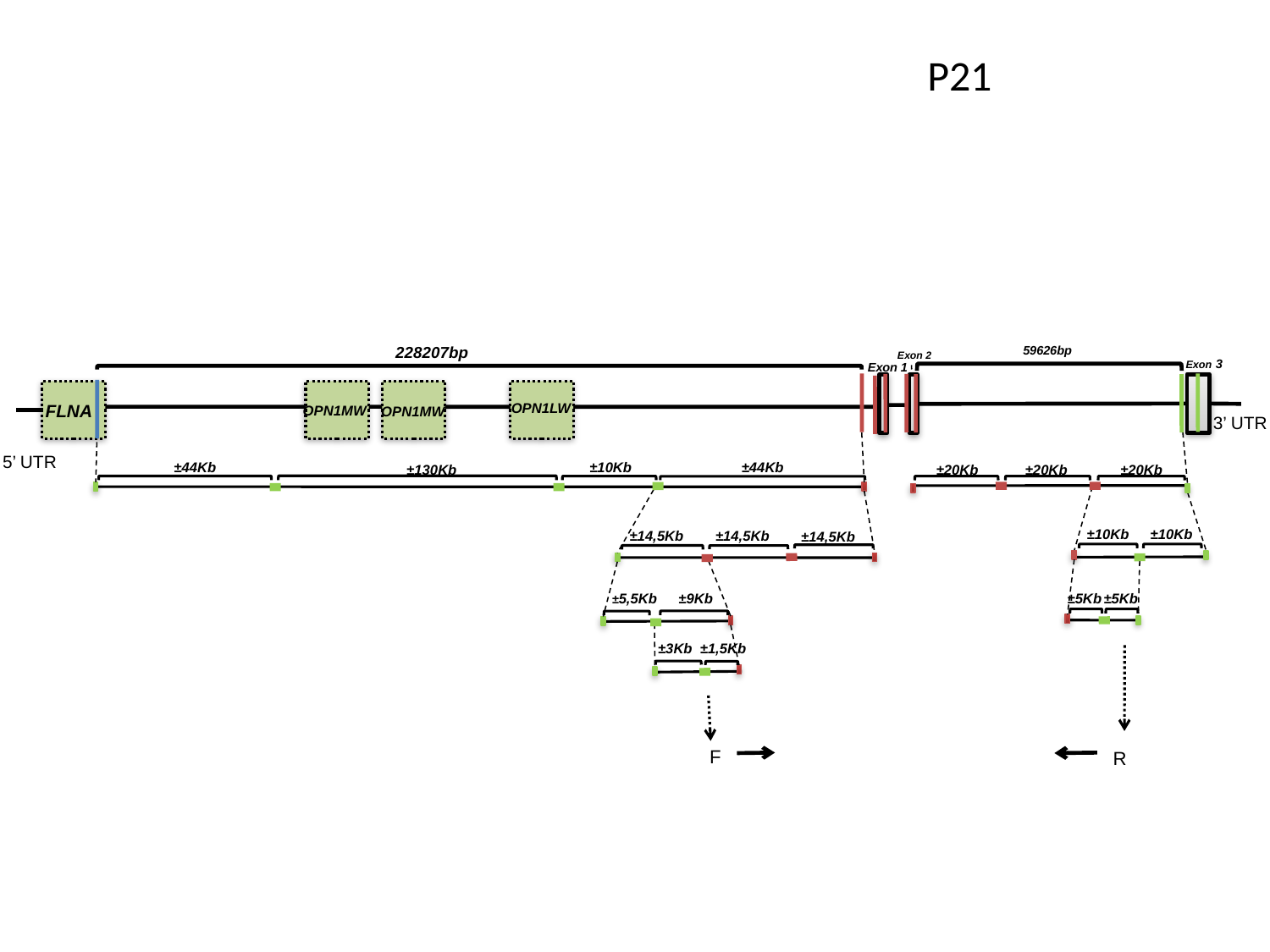

P21
±44Kb
±10Kb
±44Kb
±130Kb
±20Kb
±20Kb
±20Kb
±10Kb
±10Kb
±14,5Kb
±14,5Kb
±14,5Kb
±5,5Kb
±9Kb
±5Kb
±5Kb
±3Kb
±1,5Kb
228207bp
59626bp
Exon 2
Exon 3
Exon 1
OPN1LW
FLNA
OPN1MW
OPN1MW
3’ UTR
5’ UTR
F
R
