# Supplementary material for: Characterization of large deletions of the MECP2 gene in Rett syndrome patients by gene dosage analysis
Source: Mol Genet Genomic Med. 2019 Jun 17;7(8):e793. doi: 10.1002/mgg3.793 (PMC6687651; doi:10.1002/mgg3.793)
Supplement: Supplementary file 4 [file MGG3-7-e793-s004.docx]

| Pacient ID | Age at checklist | Onset of the first sign | Microcephaly | Sitting alone | Ambulation | Respiratory function | Epilepsy | Hands use | Onset of stereotypies | Language | Total score |
| --- | --- | --- | --- | --- | --- | --- | --- | --- | --- | --- | --- |
| P1 | 11y | 1 | 1 | 0 | 0 | 1 | 1 | 2 | 1 | 1 | 8 |
| P2 | NA | NA | NA | NA | NA | NA | NA | NA | NA | NA | NA |
| P3 | 10y | 2 | 1 | 0 | 1 | 1 | 1 | 2 | 2 | 1 | 11 |
| P4 | 17y | 2 | NA | 1 | 0 | 1 | 1 | 2 | 2 | 1 | 10 |
| P5 | 2y | 3 | 0 | 2 | 4 | 0 | 1 | 2 | 2 | 1 | 15 |
| P6 | NA | NA | NA | NA | NA | NA | NA | NA | NA | NA | NA |
| P7 | NA | NA | NA | NA | NA | NA | NA | NA | NA | NA | NA |
| P8 | NA | NA | NA | NA | NA | NA | NA | NA | NA | NA | NA |
| P9 | NA | NA | NA | NA | NA | NA | NA | NA | NA | NA | NA |
| P10 | 10y | 3 | 1 | 2 | 3 | 1 | 1 | 3 | 2 | 1 | 17 |
| P11 | 6y | 3 | 1 | 0 | 0 | 0 | 1 | 2 | 2 | 1 | 10 |
| P12 | 6y | 3 | NA | 1 | 4 | 0 | 0 | 2 | 2 | 1 | 13 |
| P13 | 4y | 3 | 1 | 0 | 3 | 1 | 1 | 2 | 2 | 1 | 14 |
| P14 | NA | NA | NA | NA | NA | NA | NA | NA | NA | NA | NA |
| P15 | 8y | 2 | 1 | 1 | 4 | 0 | 1 | 2 | 1 | 1 | 13 |
| P16 | 3y | 3 | 1 | 1 | 3 | 0 | 1 | 2 | 2 | 1 | 14 |
| P17 | 10y | 3 | 1 | 0 | 0 | 1 | 1 | 2 | 1 | 1 | 10 |
| P18 | 21y | 2 | 1 | 1 | 4 | 1 | 1 | 2 | 2 | 1 | 15 |
| P19 | 16y | 3 | 1 | 0 | 3 | 1 | 1 | 2 | 2 | 2 | 15 |
| P20 | NA | 2 | 1 | 2 | 3 | NA | 1 | 2 | 2 | 1 | 14 |
| P21 | NA | 3 | 1 | 2 | 3 | 1 | 1 | 2 | 2 | 2 | 17 |

Supplementary Data 4: The clinical score of each patient for the different characteristics measured with the Pineda score.
